# Determinants of Atrial Electromechanical Delay in Patients with Functional Mitral Regurgitation and Non-ischemic Dilated Cardiomyopathy

**DOI:** 10.15171/jcvtr.2014.019

**Published:** 2014-12-30

**Authors:** Ruken Bengi Bakal, Suzan Hatipoglu, Muslum Sahin, Mehmet Yunus Emiroglu, Mustafa Bulut, Nihal Ozdemir

**Affiliations:** Kartal Kosuyolu Heart Education and Research Hospital, Cardiology Department, Istanbul, Turkey

**Keywords:** Atrial Electromechanical Delay, Non-Ischemic Dilated Cardiomyopathy, Functional Mitral Regurgitation

## Abstract

***Introduction:*** Atrial conduction time has important hemodynamic effects on ventricular filling and is accepted as a predictor of atrial fibrillation. In this study we assessed atrial conduction time in patients with non ischemic dilated cardiomyopathy (NIDCMP) and functional mitral regurgitation (MR) and aimed to determine factors predicting atrial conduction time prolongation.

***Methods:*** Sixty five patients with non ischemic dilated cardiomyopathy who have moderate to severe MR and 60 control subjects were included in the study. In addition to conventional echocardiographic measures used to asses left ventricle and MR, atrial electromechanical coupling (time interval from the onset of P wave on surface electrocardiogram [ECG] to the beginning of A wave interval with tissue Doppler echocardiography [PA]), intra- and interatrial electromechanical delay (intra and inter AEMD) were measured.

***Results:*** The correlations between inter AEMD and left atrial (LA) size, MR volume, isovolumetric relaxation time (IVRT), deceleration time (DT), systolic pulmonary artery pressure (PAPs), E/A ratio and E/e’ were very poor. Similarly, intra AEMD was not correlated to LA size , MR volume, IVRT, DT, PAPs, E/A ratio and E/e’. However, both inter AEMD and intra AEMD had good correlation with left ventricular mass index, tenting area (TA), tenting distance (TD), coaptation septal distance (CSD), sphericity index (SI).

***Conclusion:*** Prolongation of inter and intra AEMDs were found to be well correlated with parameters reflecting left ventricular and mitral annular remodeling.

## Introduction

Atrial fibrillation (AF) is a common rhythm disorders in patients with mitral regurgitation (MR) and non-ischemic dilated cardiomyopathy (NIDCMP), moreover it is associated with increased morbidity and mortality. Increased incidence of AF is attributed to elevated left atrial (LA) pressure and fibrosis secondary to pressure and/or volume overload in the LA.^1-4^ Several studies reported that, atrial electromechanical coupling (time interval from the onset of P wave on surface electrocardiogram [ECG] to the beginning of A wave interval with tissue Doppler echocardiography [PA]) and atrial electromechanical delay (AEMD) measured by tissue Doppler imaging (TDI) were significantly longer in patients who are prone to AF.^5-7^

Functional MR is a phenomenon that occurs in the setting of adverse remodeling of the left ventricle (LV) and deformation of the mitral valve apparatus as a consequence of advanced LV systolic dysfunction.^8,9^ MR and reduced ejection fraction are associated with elevated LV and LA filling pressures which finally lead to AF, additionally they are related to adverse clinical conditions and reduced survival.^10-12^ Several echocardiographic parameters reflecting cardiac remodeling in functional MR such as tenting area (TA), tenting distance (TD), inter-papillary muscle distance (IPMD), coaptation septal distance (CSD) and sphericity index (SI) were examined recently to assess if they have a role in estimating adverse clinical outcome.^13-18^

In our study, we aimed to investigate the interatrial and intraatrial conduction times and their relation with echocardiographic parameters reflecting LV global/regional remodeling in patients with NIDCMP who have functional MR compared to normal subjects.

## Materials and methods

Sixty five patients with NIDCMP who have moderate and severe MR (35 males, 30 females; mean age: 49 ± 16 years) and 60 normal subjects (as control group, 30 males, 30 females) were included in the study. Patient group was selected from subjects with previously confirmed etiology of heart failure by coronary angiography.

Patients with a history of coronary artery disease, organic mitral valve disease, aortic valve disease, bundle branch block, atrioventricular conduction abnormalities on ECG, pericarditis, thyroid dysfunction, anemia, hypercholesterolemia, electrolyte imbalance, renal failure, pulmonary disease, echocardiographic image that was technically insufficient, patients who had electrocardiographically documented AF episode and ECG where clear-cut beginning or ending of the P wave could not be discerned were excluded from the study.

### Measurement of left ventricular indices and quantification of mitral regurgitation

A commercially available echocardiography system (Vivid 7, GE, USA) was used. The pulse wave Doppler study of the mitral and tricuspidin flows was performed from the apical four-chamber view in order to obtain the following parameters: mitral peak early (E) and peak late (A) velocities, deceleration time (DT) of E and A, mitral E/A ratio and isovolumetric relaxation time (IVRT). TDI was used to measure the early diastolic myocardial velocity (e’) at the septal and lateral mitral annulus. The E/e’ ratio was obtained using the average of septal and lateral e’. Systolic pulmonary artery pressure (PAPs) was estimated from the peak velocity of the tricuspid regurgitation obtained by continuous wave Doppler.

**Figure 1:**
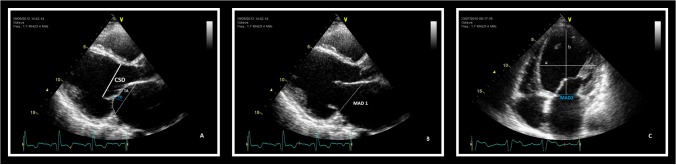
(A) Measurement of coaptation–septal distance, tenting area and tenting distance in parasternal long axis at mid-systole. (B) Mitral annulus diameter (MAD1) in parasternal long-axis view. (C) Calculating the sphericity index (SI) by the left ventricular short-to-long-axis dimension ratio (a/b) and mitral annulus diameter (MAD 2) in end-diastolic apical four-chamber view

Left ventricular volumes and EF were measured in apical four- and two-chamber views using Simpson’s method. Early diastolic mitral inflow velocity (E) was obtained at the level of the mitral leaflets in the apical four-chamber view and early diastolic tissue velocity (E’) was measured at the level of the septal mitral annulus. For determination of LV mass (LVM), the Devereux formula was used: LV mass (g): 1.04 [(LVID + PWT + IVST)3 – LVID3] – 14 (LVID = LV internal dimension PWT = posterior wall thickness IVST = interventricular septal thickness). LV mass index (LVMI) was calculated by dividing LVM by body surface area.^11^ CSD, defined as the distance between the coaptation point of the mitral valve leaflets and the septum at the hinge point of aortic valve cusp, was measured in the parasternal long-axis view at mid-systole (Figure 1A ). Mitral valve deformation was analyzed in the parasternal long-axis view at mid-systole by measuring TA measured as the area enclosed between the annular plane and the coaptation point of the mitral leaflets, and TD as the perpendicular line between the annular plane and the coaptation point of the mitral valve leaflets (Figure 1A). Mitral annular dilatation was assessed by measuring the annulus diameters in middiastolic parasternal long-axis (MAD1) and apical four-chamber (MAD2) views (Figure 1B and C). SI was calculated from the LV short-to-long-axis dimension ratio in the end-diastolic apical four-chamber view (Figure 1C). The severity of MR was determined according to regurgitant volume and effective regurgitant orifice area (EROA) using the proximal isovelocity surface area method.^19,20^ No numerical grading was performed to define the degree of MR. Instead, EROA and regurgitant volume were used as continuous variables for correlation analysis because they reflect more quantitatively the severity of the regurgitation.^14,18,21^

### Atrial Electromechanical Coupling

**Figure 2:**
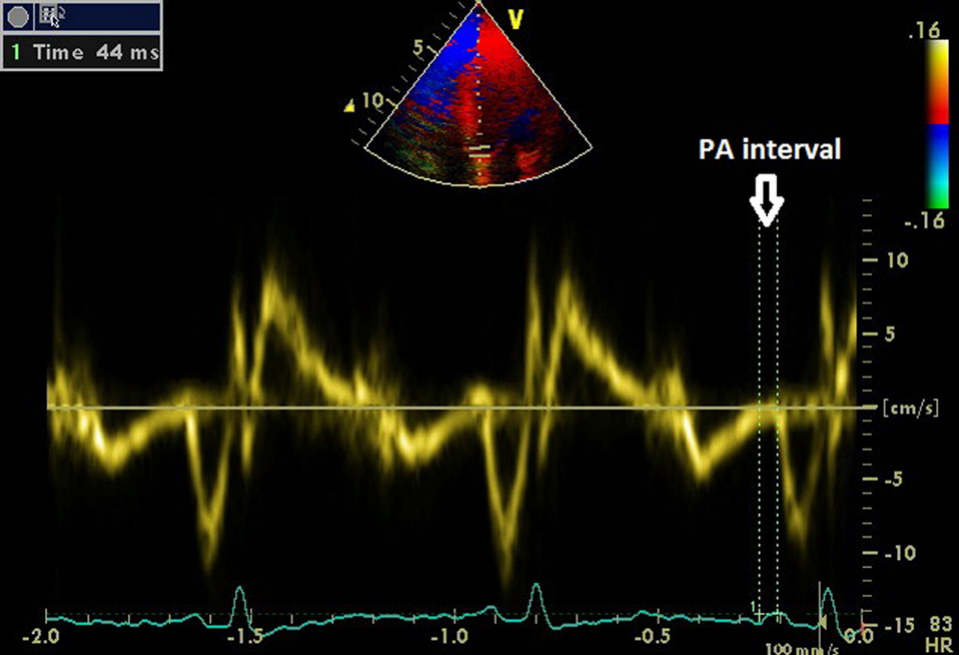
Measurement of time interval from the onset of P wave on surface electrocardiography to the beginning of Am wave (PA), with tissue Doppler echocardiography

TDI was performed by transducer frequencies of 3.5–4.0 MHz, adjusting the spectral pulsed Doppler signal filters until a Nyquist limit of 15–20 cm/s was reached, and using the minimal optimal gain. The monitor sweep speed was set at 50–100 mm/s to optimize the spectral display of myocardial velocities. In apical four-chamber view, the pulsed Doppler sample volume was subsequently placed at the level of LV lateral mitral annulus, septal mitral annulus, and right ventricular tricuspid annulus. The sampling window was positioned as parallel as possible to the myocardial segment of interest to ensure the optimal angle of imaging. The time interval (in millisecond) from the onset of P wave on surface electrocardiogram to the beginning of late diastolic wave (Am wave), which is called atrial electromechanical coupling (PA), was obtained from lateral mitral annulus, septal mitral annulus, and right ventricular tricuspid annulus and named as PA lateral, PA septum, and PA tricuspid respectively (Figure 2). Values were averaged over three consecutive beats. These values were corrected for heart rate by dividing with the square root of the R-R interval.^22^

The difference between PA lateral and PA tricuspid (PA lateral – PA tricuspid) was defined as inter AEMD, and the difference between PA septum and PA tricuspid (PA septum – PA tricuspid) was defined as intra AEMD.^6^

### Statistical Analysis

SPSS 15.0 software (SPSS Inc., Chicago, IL, USA) was used for statistical analysis. All values are presented as mean ± standard deviation. Normal distribution was tested with Kolmogorov-Smirnov test .Values between different groups were compared using the independent-samples student t-test. The χ^2^ test was used to assess differences between categorical variables. The relationship between parameters was determined using the Pearson’s coefficient of correlation. A two- tailed P value of <0.05 was considered significant for all tests.

## Results

The clinical characteristics and conventional echocardiographic parameters of patients included in the study are given in the Table 1 and 2. Control and patient groups were similar regarding age, gender, body mass index, body surface area (Table 1). There were significant differences in diastolic filling parameters between control and study group. E/A ratio (1.8 ± 1.1 vs. 1.1 ± 0.4, P=0.001), E/e’ (23 ± 2 vs. 11 ± 4, P=0.001) were greater in study group and IVRT (74 ± 26 vs. 98 ±11, P= 0.001), DT (166 ± 54 ms vs. 175 ± 24 ms, P=0.001) were longer in control group suggesting diastolic dysfunction, especially restrictive pattern was very prevalent among patients with functional MR (All p values were <0.001). Systolic pulmonary artery pressure (PAPs) (50 ± 13 vs. 23 ± 5 mmHg, P=0.01) and LA antero-posterior diameter (4.4 ± 0.6 vs. 2.9 ± 0.5 cm, P=0.001) were higher in study group. Mitral annular area (9 ± 3.1 vs. 5 ± 1.3 cm², P=0.01), LVMI (132 ± 40 vs. 83 ±24 g, P=0.01), TD (1.3 ± 0.4 vs. 0.3 ± 0.1 cm, P=0.001), CSD (3.3 ± 0.5 vs. 1.3 ± 0.3 cm, P=0.001), SI (0.6 ± 0.18 vs. 0.38 ± 0.14, P=0.001) and TA (2.4 ± 0.8 vs. 0.8 ± 0.3 cm², P=0.001) were found to be greater in study group as expected (P<0.001) (Table 2). PA lateral, PA septal, PA tricuspid and both inter AEMD and intra AEMD were prolonged in patients with functional MR compared to controls (Table 2).

**Table 1:** Demographic characteristics of the study patients

**Variables**	**Study** ** (n=65)**	**Control** ** (n=60)**	** P**
Age (years)	48±15	45 ±17	NS
Gender (male/female)	35/30	30/30	NS
BSA (m^2^)	1.8±0.4	1.9 ±0.6	NS
BMI (kg/m^2^)	27 ±5.3	29 ±6.4	NS

Data are presented as mean ± standard derivation. BSA: body surface area; BMI: body mass index

**Table 2:** Echocardiographic characteristics of the study patients

**Variables**	**Study** **(n=65)**	**Control** **(n=60)**	**P**
E/A ratio	1.8 ± 1.1	1.1 ± 0.4	0.001
E/e'	23 ± 2	11 ± 4	0.001
IVRT(msc)	74 ± 26	98 ± 11	0.001
DT (msc)	166 ± 54	175 ± 24	0.001
PAPs (mmHg)	50 ± 13	23 ± 5	0.001
LA (cm)	4.4 ± 0.6	2.9 ± 0.5	0.001
ROA (cm^2^)	0.45 ± 0.21	-	
Vena contracta (cm)	0.6 ± 0.24	-	
MR volume (ml)	60 ± 33	-	
Mitral annular area (cm^2^)	9 ± 3.1	5 ± 1.3	0.001
Mass index (g/m^2^)	132 ± 40	83 ± 24	0.001
Tenting distance (cm)	1. 3 ± 0.4	0.3 ± 0.1	0.001
Coaptation septal distance (cm)	3.3 ± 0.5	1.3 ± 0.3	0.001
Sphericity index	0.6 ± 0.18	0.38 ± 0.14	0.001
Tenting area (cm^2^)	2.4 ± 0.8	0.8 ± 0.3	0.001
PA lateral C (msc)	81 ± 24	63 ± 13	0.001
PA septal C(msc)	51 ± 17	37 ± 11	0.001
PA tricuspit C(msc)	36 ± 13	21 ± 9	0.001
Interatrial EMD(msc)	37 ± 12	21 ± 7	0.001
Intraatrial EMD(msc)	18 ± 5	9 ± 5	0.001
Left ventricular EF,%	32 ± 8	62 ± 10	0.001

Data are presented as mean ± standard derivation. E: early diastolic mitral inflow velocity; A: late diastolic mitral inflow velocity; e’: early diastolic mitral annular velocity. IVRT: isovolumetric relaxation time; DT: deceleration time; PAPs: systolic pulmonary artery pressure; LA: left atrium; ROA: regurgitant orifice area; MR: mitral regurgitation; PA: atrial electromechanical coupling; AEMD: atrial electromechanical delay; EF: ejection fraction

**Figure 3:**
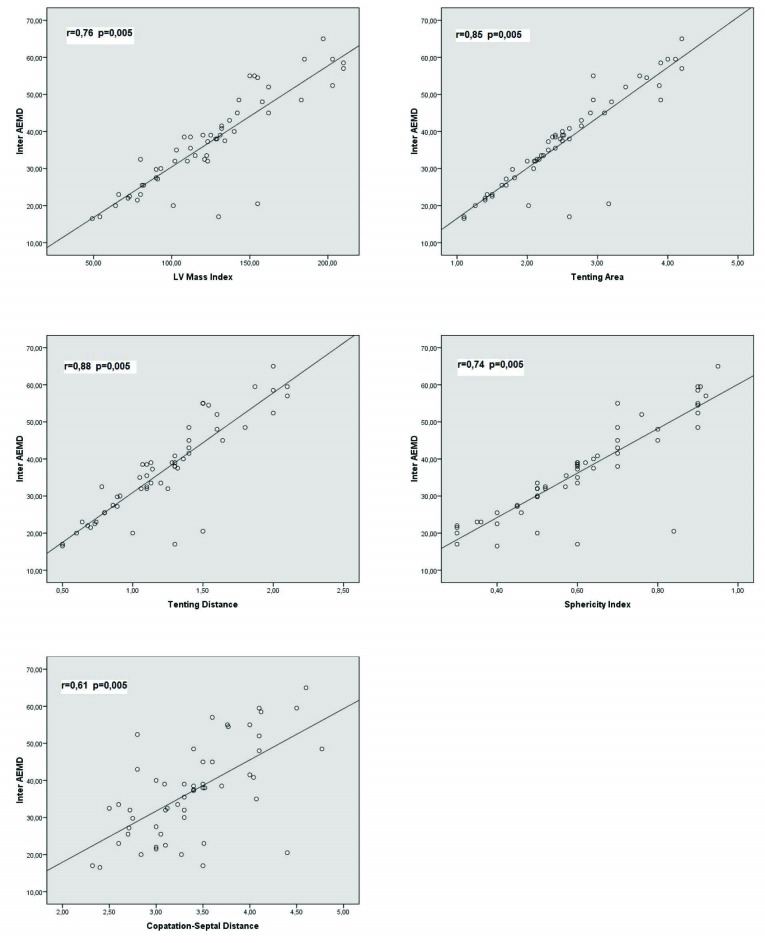
Correlation between interatrial electromechanical delay and left ventricular mass index, tenting area, tenting distance, coaptation septal distance, sphericity index.

**Figure 4:**
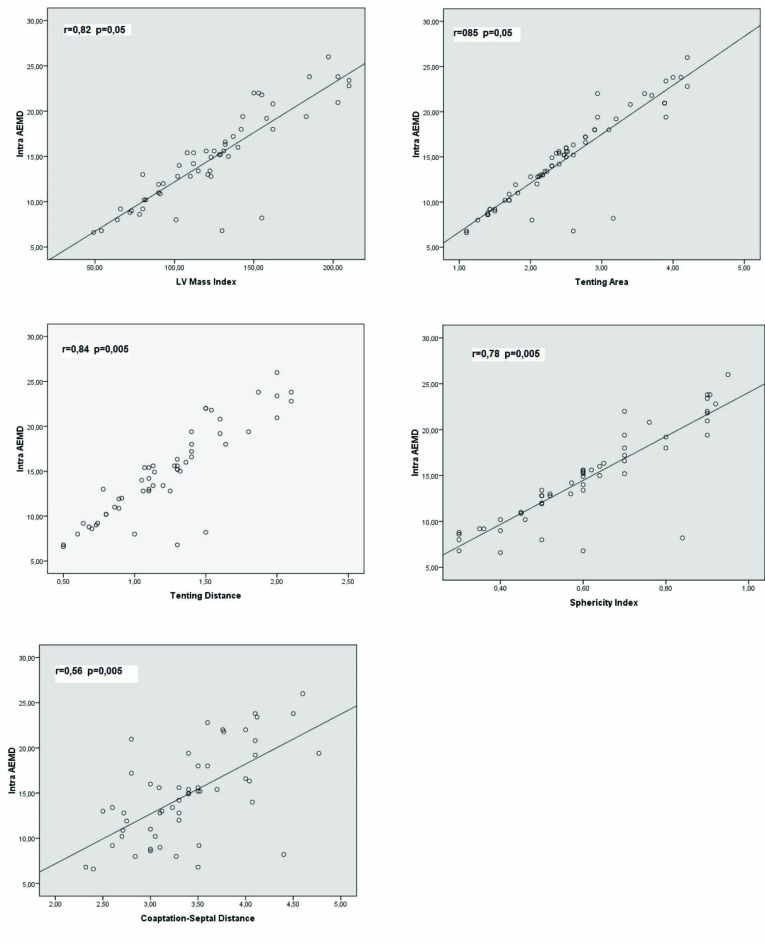
Correlation between intraatrial electromechanical delay and left ventricular mass index, tenting area, tenting distance, coaptation septal distance, sphericity index

Correlation analysis was performed to determine the parameters that were related to prolonged electromechanical delay in patients with functional MR. The correlations between inter AEMD and LA size (r= 0.34, P=0.006), MR volume (r=0.35, P=0,023), IVRT (r= -0.07, P= 0.5), DT ( r= -0.147, P= 0.2), PAPs (r=0.3, P= 0.05), E/A ratio (r=0.23, P= 0.065) and E/e’ (r=0.147, P= 0.242) were very poor. Similarly there were no correlation or a very poor one between intra AEMD and LA size (r=0.34, P=0.006), MR volume (r= 0.29, P=0.04), IVRT (r= -0.7, P=0.53), DT (r=-0.21, P= 0.07), PAPs (r=0.4, P=0.05), E/A ratio (r=0.28, P= 0.05) and E/e’ (r=0.24, P= 0.3). Conversely, correlations between inter AEMD and LVMI (r=0.76, P=0.005), TA (r= 0.85, P=0.005), TD ( r=0.88, P=0.005 ), CSD ( r= 0.61, P=0.005), SI (r=0.74, P=0.005) (Figure 3) and correlations between intra AEMD and LVMI (r=0.82, P=0.05), TA (r=0.85, P=0.005 ), TD (r=0.84, P=0.005), CSD (r= 0.56, P= 0.005), SI (r= 0.78, P=0.005) (Figure 4) were rather good.

## Discussion

AF is also one of the most commonly encountered rhythm disorders in patients with MR and is directly associated with increased mortality and morbidity. Increased incidence of AF is attributed to elevated LA pressure and fibrosis secondary to pressure overload in the left atrium.^1-4^ Fibrosis and enlarged LA tissue prolong and divert the propagation of action potential in LA tissue, and cause multiple micro reentries resulting in AF.^4-7,23,24^

The prolongation of atrial conduction times were shown in many clinical circumstances such as amyloidosis, coronary artery disease, paroxysmal AF, mitral stenosis, rheumatoid arthritis, Behçet’s disease and NIDCMP.^26-35^, It was detected in our study that intra/inter AEMD were prolonged in patients with NIDCMP who have moderate to severe functional MR compared to control group. The prolongation of intra- and inter AEMD and the inhomogeneous propagation of sinus impulses are well-known electrophysiological characteristics of the atria prone to fibrillation.^5-7^ This prolongation in inter/intra AEMD corresponds to the increased AF incidence in patients with NIDCMP who have functional moderate to severe MR. In literature, our study is the first to report that both inter and intra AEMD are prolonged in this patient group.

Several studies have shown that LV local and global remodeling, papillary muscle dysfunction and displacement, mitral annular dilatation and intraventricular dyssynchrony contribute independently to regurgitation of the anatomically normal mitral valve. It has also been shown that global remodeling associated with chamber enlargement, increased sphericity, and several echocardiographic parameters such as TA, TD, IPMD, and CSD have been shown to reflect the geometric alterations in the mitral valve apparatus and to give accurate estimations of prognosis in patients with NIDCMP.^13-18^ But the association between AEMD and these prognostic parameters is not well examined. So, the next aim of the study was to explore the effects of hemodynamic parameters and the parameters that reflect the left ventricular remodeling on AEMD and indirectly on the incidence of AF.

As we examine the effects of hemodynamic and left ventricular remodeling parameters on AEMD, no association was detected between AEMD and indices reflecting left ventricular diastolic performance (E/A, E/e’, IVRT, DT), PAPs and parameters reflecting the degree of MR (vena contracta, MR volume). Good correlations were detected between AEMD and mitral annular area, LVMI, CSD, SI, TA and TD which are the determinants of left ventricular dilation and remodeling, as well as deformation of mitral annular apparatus.

These results indicate that hemodynamic determinants such as the degree of diastolic dysfunction, the degree of MR, and PAPs have no important effects on AEMD in patients with NIDCMP who have mild to moderate functional MR. Because hemodynamic findings are relatively labile and may change from time to time and also in response to therapy, it is considered that they are not able to predict prolongation in AEMD which were mainly caused by chronic alterations in tissue architecture such as fibrosis and remodeling. As mitral annular area, LVMI, CSD, SI, TA and TD are the determinants of cardiac remodeling and dilatation caused by both the disease itself and functional MR, they are considered to reflect the disease severity and prolongation in AEMD more precisely.

The fact that the previous studies blamed chronic fibrotic changes and LA remodeling in the emergence of AF^4,9^ supports our idea that parameters reflecting global/ regional remodeling are better than hemodynamic parameters in predicting the prolongation of AEMD in patients with NIDCMP who have moderate to severe functional MR.

Both intra/inter AEMD were prolonged in patients with NIDCMP who have functional MR compared to normal subjects. In this patient group, prolongation of AEMD times were found to be well correlated with parameters reflecting left ventricular and mitral annular remodeling. However, the correlations between AEMD times and parameters reflecting hemodynamic status such as diastolic filling parameters, PAPs, severity of MR were absent or very scant.

## Ethical issues

Written informed consent was obtained from each subject and our institutional ethics committee approved the study protocol.

## Competing interests

Authors declare no conflict of interests in this study.
